# Grossesse intra murale à propos d'un cas

**DOI:** 10.11604/pamj.2015.21.217.7274

**Published:** 2015-07-24

**Authors:** Kofi-Mensa Savi de Tové, Kabibou Salifou, Rachidi Sidi Imorou, Olivier Biaou, Vicentia Boco

**Affiliations:** 1 Faculté de Médecine, Université de Parakou, Bénin; 2 Faculté des Sciences de la Santé, Université d'Abomey Calavi, Bénin

**Keywords:** Grossesse extra-utérine, grossesse intra-murale, échographie, métrorragies, ectopic pregnancy, intramural pregnancy, echography, metrorrhagia

## Abstract

La grossesse intra-murale est la variété la plus rare de grossesse extra-utérine. Il s'agit de la localisation de l’œuf dans l’épaisseur du myomètre. En cas de retard diagnostic, l’évolution peut être catastrophique avec rupture utérine et hémorragie cataclysmique. L’échographie permet dans certains cas un diagnostic pré opératoire. Les auteurs rapportent un cas survenu chez une patiente aux antécédents de curetage.

## Introduction

Les grossesses extra-utérines sont une cause redoutée de métrorragies du premier trimestre. Elles correspondent à la nidation et au développement de l’œuf en dehors de la cavité utérine. La localisation intra-murale est rare et correspond à la nidation de l’œuf dans la profondeur du myomètre au sein d'une cavité ne communiquant ni avec la cavité utérine ni avec la lumière tubaire [1]. Elle est d’évolution imprévisible avec risque d'hémorragie cataclysmique par rupture utérine en absence d'un diagnostic et d'une prise en charge précoce [2]. Nous rapportons ici le cas d'une grossesse intra murale chez une patiente aux antécédents de curetage.

## Patient et observation

**Figure 1:**
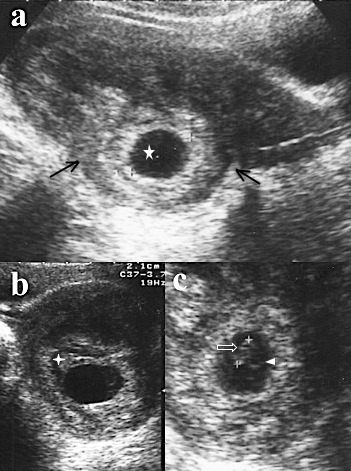
Images échographiques de la grossesse intra murale; a): coupe sagittale: sac ovulaire (étoile) de 22,3mm avec une double couronne trophoblastique bien visualisée, la ligne cavitaire est visible sans réaction déciduale marquée, à noter au pôle supérieur du sac un petit amas échogène (étoile à cinq branche creuse) évoquant un petit caillot, il existe une nette déformation des contours utérins (entre les 2 flèches noires) ainsi qu'un net amincissement du myomètre en regard du sac (entre 3 et 1 mm); b): coupe transversale, anéchogénicité soulignant le sac ovulaire et signant une hématométrie; c): coupe oblique, embryon de 7,1mm soit 6SA+4 jours (flèche blanche creuse) et vésicule ombilicale (tête de flèche blanche)

Il s'agit d'une patiente de 32 ans nullipare chez qui une échographie pelvienne est réalisée pour des douleurs pelviennes sans métrorragies sur une aménorrhée de deux mois. L'interrogatoire retrouve un antécédent de fausse couche provoquée par curetage il y a 3 ans. L’échographie pelvienne par voie sus pubienne a permis de poser le diagnostic de grossesse ectopique intra-murale de 6SA+4 jours avec un embryon vivant (Figure 1). Au décours de l'examen échographique, la survenue de métrorragies abondantes faites de sang rouge vif imposa une laparotomie en urgence. Le diagnostic de grossesse intra murale compliquée de rupture utérine fut posé en per opératoire. Une hystérectomie fut réalisée avec des suites opératoires simples.

## Discussion

La grossesse intra murale est une entité extrêmement rare représentant moins de 1% des grossesses extra-utérines [3]. Jusqu'en 2013, seuls 53 cas ont été rapportés dans la littérature. Ces grossesses surviennent en cas de cicatrices myométriales communicant avec la cavité utérine. Il peut s'agir de cicatrices de césariennes ou de myomectomie, de cavités adénomyomateuses ou après curetage comme dans notre cas [1]. Le principal risque de cette localisation est la rupture utérine avec hémorragie cataclysmique [4]. Cliniquement elle se traduit avant la survenue de la rupture par l'association d'une aménorrhée, de douleurs pelviennes, de métrorragies et d'un TBG positif. Ces signes sont ceux de toute grossesse ectopique ou d'un avortement spontané. Cette absence de spécificité des signes cliniques fait tout l'intérêt de l'imagerie. L’échographie pose le diagnostic en visualisant un sac ovulaire excentré et une cavité utérine vide ou siège d'une hématométrie comme dans notre cas (Figure 1, b). Selon Luo et al il existerait trois formes échographiques de grossesse intra murale: la forme kystique, la forme nodulaire et la forme rompue. Dans notre cas il s'agit d'une forme kystique embryonnée [5]. Le diagnostic reste difficile à l’échographie mais dans notre cas, l'amincissement myométrial associé à la déformation des contours utérins faisait fortement suspecter le diagnostic (Figure 1, a). La mise évidence de l'embryon a permis d’écarter les classiques diagnostics différentiels que sont un myome en transformation kystique et une tumeur trophoblastique (Figure 1, c). La localisation sus-isthmique loin des cornes utérines a permis d’éliminer une grossesse interstitielle chez notre patiente (Figure 1, a). Le doppler en visualisant l'hypervascularisation et les reconstructions 3D en permettant une meilleure localisation du sac améliorent le diagnostic échographique. L'IRM est d'un apport certain en cas de doute diagnostic [4, 6]. Le traitement est le plus souvent chirurgical, parfois conservateur par laparotomie [4] ou laparoscopie [7]. Dans les cas vus tôt un traitement médical par injection de methothrexate donne de bons résultats [3].

## Conclusion

La grossesse intra murale est la plus rare des grossesses ectopiques. L’échographie peut permettre un diagnostic pré opératoire avant la rupture utérine.

## References

[1] Kirk E, McDonald K, Rees J, Govind A (2013). Intramural ectopic pregnancy: a case and review of the literature. Eur J Obstet Gynecol Reprod Biol..

[2] Badr S, Ghareep A-N, Abdulla LM, Hassanein R (2013). Ectopicpregnancy in uncommon implantation sites. Egypt J Radiol Nucl Med..

[3] Ong C, Su L-L, Chia D, Choolani M, Biswas A (2010). Sonographic diagnosis and successful medical management of an intramural ectopic pregnancy. J Clin ultrasound..

[4] Fadhlaoui A, Kharouf M, Nouira K, Chaker A, Zhioua F (2011). Ruptured intramural pregnancy with myometrial invasion treated conservatively. Case Rep Obstet Gynecol..

[5] Luo Z, Zhou P, Gao F, HE W, Luo S (2010). Diagnosis of intramural pregnancy by endoluminal color Doppler ultrasonography and review of the literature. J South Med Univ..

[6] Bouzari Z, Keshani M, Yazdani S, Barat S, Zinalzadeh M (2010). Intramural pregnancy. J Obstet Gynaecol..

[7] Nabeshima H, Nishimoto M, Utsunomiya H, Arai M, Ugajin T, Terada Y (2010). Total laparoscopic conservative surgery for an intramural ectopic pregnancy. Diagn Ther Endosc..

